# Insular functional connectivity in autistic and non-autistic development

**DOI:** 10.1016/j.biopsycho.2025.109043

**Published:** 2025-04-30

**Authors:** Alisa R. Zoltowski, Michelle D. Failla, Fiona Wu, Caitlin A. Convery, Brianna Lewis, Neil D. Woodward, Baxter P. Rogers, Carissa J. Cascio

**Affiliations:** a Life Span Institute, University of Kansas, Lawrence, KS, USA; b Department of Psychiatry and Behavioral Sciences, Vanderbilt University Medical Center, Nashville, TN, USA; c Frist Center for Autism and Innovation, Vanderbilt University, Nashville, TN, USA; d College of Nursing, Ohio State University, Columbus, OH, USA; e Vanderbilt University, Nashville, TN, USA; f Department of Radiology and Radiological Sciences, Vanderbilt University Medical Center, USA; g Vanderbilt University Institute of Imaging Science, Nashville, TN, USA; h Department of Psychology, University of Kansas, Lawrence, KS, USA

**Keywords:** Interoception, Resting state, Functional connectivity, Autism, Insula, Development

## Abstract

**Background:**

There is evidence for altered interoceptive processing in individuals diagnosed with autism, compared to non-autistic individuals. At a neural level, functional and structural connectivity of interoceptive cortices may differ in autism, though developmental patterns of these differences are unclear as well as how these patterns may vary by subregion within the insular cortex. To better understand the roles of autism, age, and subregion in interoceptive connectivity patterns, we used a cross-sectional approach to examine interoceptive functional connectivity across individuals spanning a wide age range.

**Methods:**

N = 59 autistic individuals (ages 7–54) and N = 71 non-autistic individuals (ages 7–51) completed a resting-state functional magnetic resonance imaging scan. From these scans, we analyzed seed-based functional connectivity of insula subregions (posterior, middle, and anterior) by hemisphere. We analyzed associations with age, group, and interoceptive self-reported experiences, as measured in a subset of individuals who completed the Body Perception Questionnaire.

**Results:**

We found that with age, primary interoceptive cortex showed decreased functional coupling with subcortical regions such as the thalamus and increased coupling with multimodal parietal regions. Functional connectivity within key interoceptive areas was decreased in those with increased reported body awareness. Differences between the autistic and non-autistic groups were minimal, with a single finding of heightened connectivity in autism between left posterior insula and lateral occipital cortex.

**Conclusions:**

These findings shed light on potential developmental shifts in how interoceptive processing is balanced between lower-order and higher-order areas. Further, they provide background for how autistic patterns of interoceptive processing may be considered relative to age.

## 1. Introduction

Altered interoception, i.e., the sensory perception of cues signaling biological needs, has been of growing interest in clinical conditions (Khalsa et al., 2018), including its relation to the other sensory processing differences that are among the core features of autism (American Psychiatric Association, 2013; Williams et al., 2022). However, interoception presents an interesting paradox: these are among the most crucial cues for our survival, yet we are often not consciously aware of them (Ainley et al., 2016; Allen et al., 2022; Barrett & Simmons, 2015; Craig, 2002; Kleckner et al., 2017). Barrett and Simmons (2015) propose that efficient design of the neural circuits for interoception provide an explanation for this paradox. These circuits efficiently meet the body’s needs by continually anticipating those needs based on past experiences and current state (Barrett & Simmons, 2015; Paulus & Stein, 2006). In this model, successfully predicted physiological changes may be filtered from conscious awareness, but unpredicted changes reach conscious awareness so that the individual may respond to and learn from the body’s newly perceived need. Thus, the brain systems that underlie the anticipation of interoceptive signals may reveal more about altered interoceptive experiences than behavioral measures alone.

**Fig. 1.**
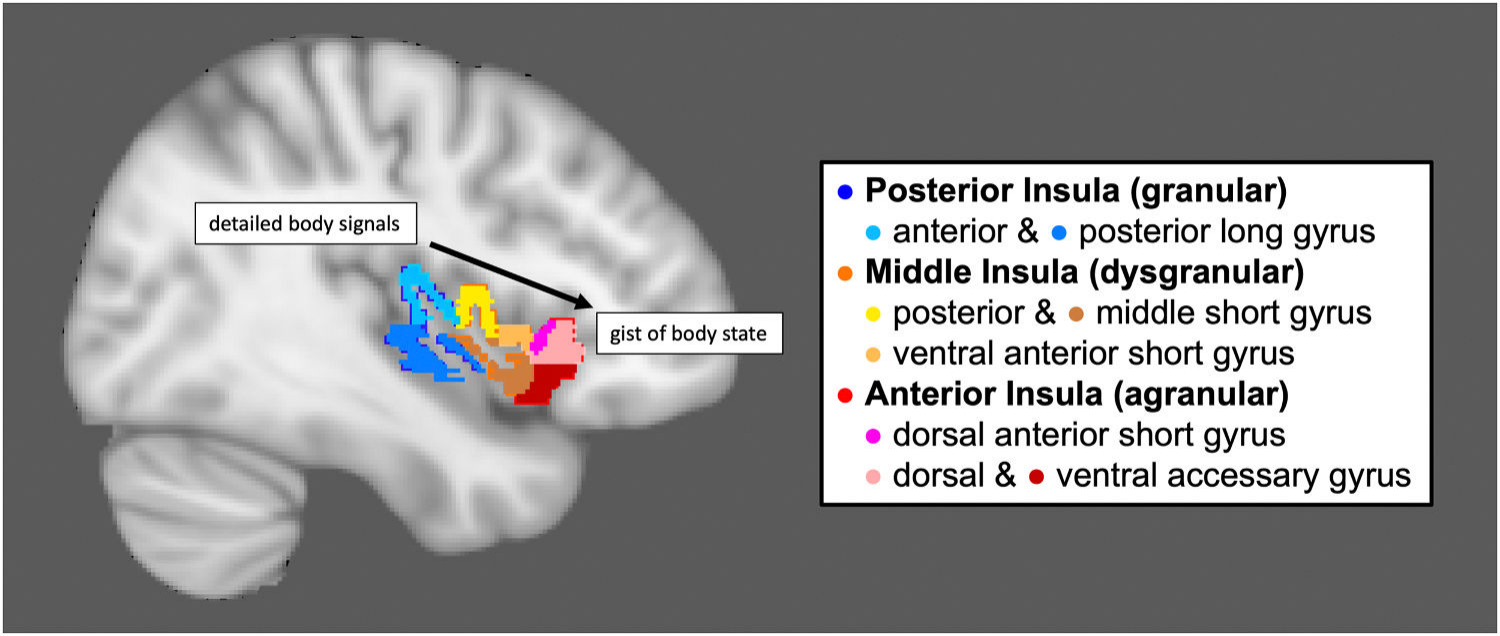
Subregions of the human insula. Subregions of the human insula are shown for both detailed (8-subregion) and major (posterior, middle, anterior) categorizations. The figure legend shows corresponding granularity (granular, dysgranular, or agranular) of each subregion.

The primary interoceptive network includes specialized subregions of insular cortex that coordinate to sense, anticipate, and contextualize bodily sensations. Cytoarchitectural analysis of insular cortex reveals eight distinct subregions (Farb et al., 2013), commonly grouped into three major subdivisions that each map to a broader function: the posterior (PI), middle (MI), and anterior insula (AI, Fig. 1). The granular PI receives direct input from subcortical regions, as the first site of conscious awareness of bodily sensations. The dysgranular MI receives interoceptive information from PI as well as diverse multimodal sensory inputs, which may then be associated together. Information that reaches the agranular AI reflects an abstracted summary of bodily state as predicted by and interpreted within the current environmental context (Barrett & Simmons, 2015; Kleckner et al., 2017). Though many models do not distinguish between left and right insular processing, Bud Craig’s (2002, 2005, 2008) neuroanatomic work suggests that the right insula specializes in controlling the sympathetic nervous system and the left insula specializes in controlling the parasympathetic nervous system.

Autistic individuals self-report difficulty detecting and interpreting their interoceptive signals, yet the mechanistic basis of these differences remains unclear (DuBois et al., 2016; Failla et al., 2020; Fiene & Brownlow, 2015; Garfinkel et al., 2016; Palser et al., 2018; Quadt et al., 2021; Trevisan, Parker, et al., 2021; Williams et al., 2022). Of the three major insular subdivisions, the AI has been the most studied in autism (Di Martino et al., 2014; Uddin, 2015; Uddin & Menon, 2009), as a hub of the salience network. Uddin (2015) reviews the prominence of AI connectivity differences found between autistic and non-autistic samples, though the specific regions of identified differences have been heterogeneous between samples (Uddin, 2015). One important factor that may explain this heterogeneity is development (Nomi et al., 2019; Uddin et al., 2013). Nomi et al.’s (2019) model suggests a shift in autism from a hyper- connected AI in childhood to hypo- connectivity in adolescence and adulthood, implicating over-proliferation followed by accelerated pruning of AI connections (Hua & Smith, 2004; Innocenti & Price, 2005; Katz & Shatz, 1996).

Less is known about the PI and MI in autism. Failla et al. (2017) reported differences in the white matter connecting the PI to AI in autistic compared to non-autistic individuals, suggesting altered communication along this pathway. Studies of PI and MI functional connectivity in autism have mostly been conducted in small adolescent samples and have reported reduced PI connectivity with somatosensory (Ebisch et al., 2011) and visual areas (Francis et al., 2019). Xu et al. (2018) reported differences that localized to the MI but not PI. Looking across hemispheres, Ebisch et al. (2011) reported left-lateralized PI-somatosensory hypoconnectivity. This finding could implicate an imbalance favoring sympathetic input in autonomic subsystems according to Craig’s laterality model, in line with broad findings of higher rates of anxiety in autism (Kent & Simonoff, 2017). Further, one functional homogeneity study found evidence that the MI constitutes a larger functional region in autistic compared to non-autistic individuals (Yamada et al., 2016). Together, these findings suggest distinct roles of the PI and MI in autistic interoception and how these might relate to factors such as stress and anxiety.

Developmental trajectories of insula connectivity differences have only been examined in a limited number of these studies. These may further help synthesize findings, as with patterns of AI connectivity with age (Nomi et al., 2019; Uddin et al., 2013) and other known developmental changes in interconnected regions such as the amygdala (Gabard-Durnam et al., 2014). One recent study suggests these networks continue to evolve from young to later adulthood (Dobrushina et al., 2024), though how these patterns connect to childhood development is less well known. Francis et al. (2019) found widespread amelioration of PI hypoconnectivity with age in their adolescent sample, narrowing the difference between autistic and neurotypical samples at older ages. Similar amelioration is found in behavioral (Feldman et al., 2018) and neural (Dunham et al., 2023) studies of multisensory processing in autism, providing further context for how communication between different sensory brain regions may mature in autistic compared to non-autistic individuals.

However, before examining how insula development across these three subregions diverges in autism, general developmental patterns across the three subregions must be more completely understood. Thus, in this sample we examined age-related trends in posterior, middle, and anterior insula functional connectivity across our full sample. Though heterogeneity in autistic development is also important to consider, combining our full sample allows increased power to detect developmental patterns that are robust to neurotype. While exploratory in nature, predictive coding models of interoception might suggest that the areas that functionally couple with these regions become increasingly diverse over the course of development, which may further depend on each individuals’ environmental experiences. We further expected that these patterns might vary by insula functional subdivision, perhaps staying the most stable within the primary sensory posterior insula. We also examined preliminary group-based differences in insular functional connectivity in autistic versus non-autistic individuals. Lastly, to contextualize the functional role of the insula in interoception, we examined dimensional relationships with self-reported body awareness.

## 2. Methods and materials

### 2.1. Participants

**Fig. 2.**
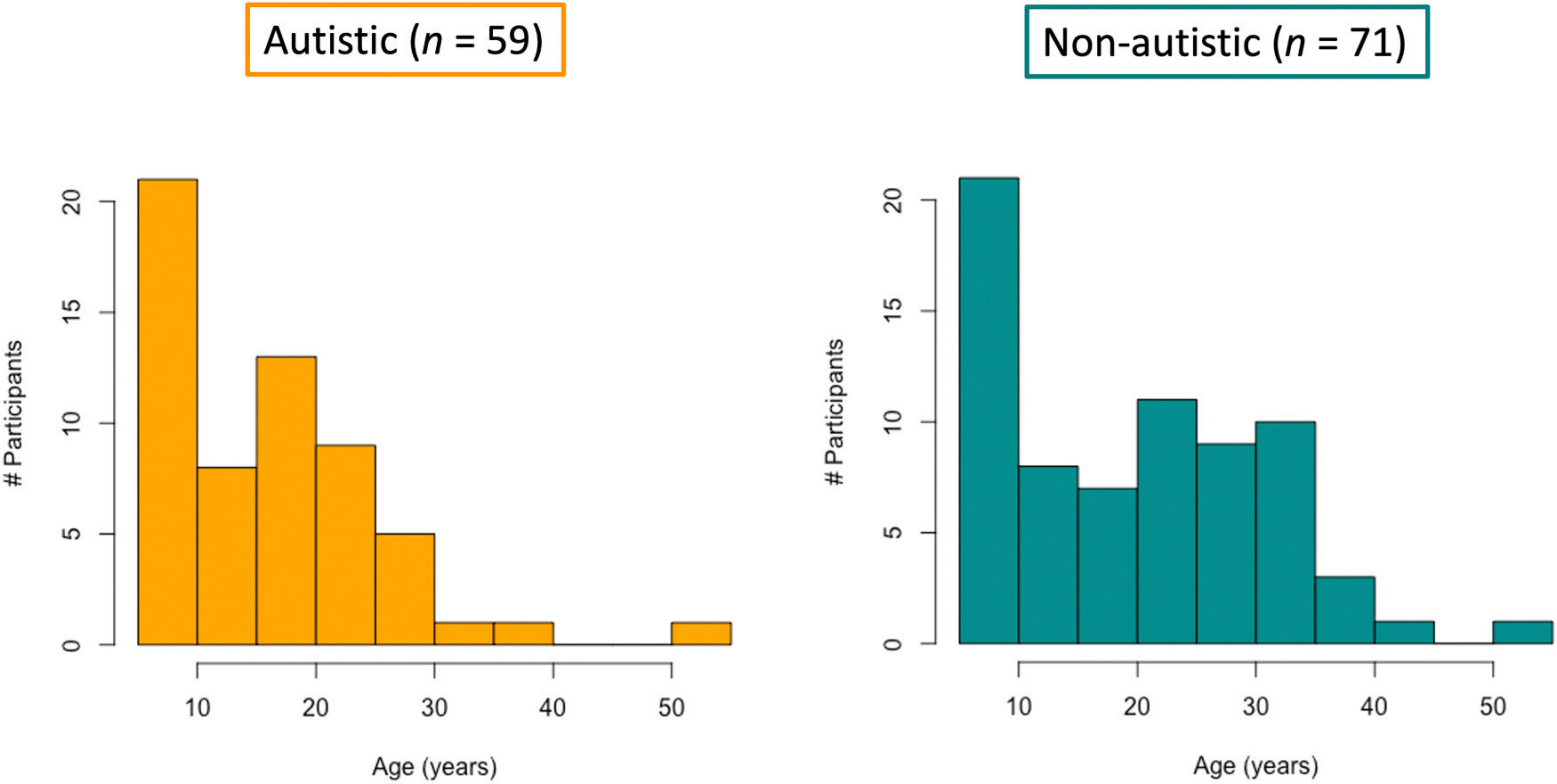
Distribution of sample ages by diagnostic group. Histograms of participant age per group (Autistic versus Non-Autistic) are plotted, as number of participants (y-axis) per five-year age groups (x-axis). Autistic group is in orange and non-autistic group is in teal.

The full sample comprised *n* = 59 autistic individuals (AUT, ages 7–54) and *n* = 71 non-autistic individuals (N-AUT, ages 7–53). See Table 1 and Fig. 2 for participant characteristics. Individuals included in the autism group had diagnoses confirmed by a licensed clinical psychologist, using the Autism Diagnostic Observation Schedule, General (ADOS-G, Lord et al., 2000) or Second Edition (ADOS-2, Lord et al., 2012), and clinical judgment. Individuals were recruited across multiple parent functional magnetic resonance imaging (fMRI) cohorts; prior reports of functional data have been published in Cascio et al. (2014) and Failla et al. (2020). All study participants provided informed consent/assent and the Vanderbilt University Institutional Review Board approved all parent studies. REDCap electronic data capture tools, hosted at Vanderbilt University, were used to collect and manage study data (Harris et al., 2009, 2019).

Individuals were excluded from each parent MRI study for having any contraindications to MRI scanning (see Supplemental Information), with final decisions made by staff MRI technicians, or if intellectual quotient (IQ) was below 70. Of an initial *n* = 68 autistic and *n* = 79 non-autistic individuals who completed resting state scans across cohorts, *n* = 9 autistic and *n* = 8 non-autistic participants were excluded due to failed structural (*n* = 2 AUT, *n* = 3 N-AUT excluded) or functional (*n* = 7 AUT, *n* = 5 N-AUT excluded) quality assurance, as defined below.

Participants were included in interoceptive self-report analyses if they had completed the Body Perception Questionnaire (BPQ, Porges, 1993). The subset of the sample included in BPQ analyses were *n* = 32 autistic and *n* = 24 non-autistic participants. The BPQ is a questionnaire designed to measure reported awareness of bodily sensations in everyday contexts. Scores were analyzed using the short form items as published in Cabrera et al. (2018), though participants who participated prior to the short form publication received the full set of items. The “Body Awareness” subscale was analyzed given its common use in studies of interoception in autism (Williams et al., 2022). The “Awareness” subscale short form shows an estimated internal consistency of omega = 0.96 in a large American online sample, as analyzed categorically (yes/no) in Cabrera et al. (2018). Any language or terms in the questionnaire that were confusing to youth and/or autistic participants was simplified by the study team.

### 2.2. Image collection

To retain accuracy in reporting, the following Materials and Methods sections (*Image collection, MRI Processing, Structural preprocessing,* and *Functional Preprocessing*) have been retrieved closely from the first author’s unpublished dissertation, as can be found at: http://hdl.handle.net/1803/18595.

Anatomical and resting state functional images were acquired via one of three protocols:

Protocol 1 (*n* = 27 autistic, *n* = 24 non-autistic, ages 7–35):

- High-resolution T1-weighted anatomical images were acquired via sagittal slices with 1 mm^3^ voxel resolution, TR= 9.0 ms, TE= 4.6 ms, flip angle= 8°, and acquisition matrix= 256 × 256 × 170.
- Resting state images were acquired using an echo planar imaging (EPI) sequence (3 mm^3^ isotropic voxels, TR=2 s, flip angle 90) for approximately 6 min 46 s duration (203 volumes).

Protocol 2 (*n* = 29 autistic, *n* = 36 non-autistic, ages 7–54) and Protocol 3 (n = 3 autistic, n = 11 non-autistic, ages 9–44):

- High-resolution T1-weighted anatomical images were acquired via sagittal slices with 1 mm^3^ voxel resolution, TR= 8.0 ms, TE= 3.7 ms, flip angle= 7°, and acquisition matrix= 256 × 256 × 170.
- Resting state images were acquired using an echo planar imaging (EPI) sequence (3×3×4 mm voxels, TR=2 s, flip angle 79, and acquisition matrix 80 ×80 × 28) for approximately 6 min 46 s duration (203 volumes).

Though Protocols 2 and 3 had identical acquisition parameters, these were considered separate covariates in all analyses due to differences in the preceding task (interoception task or special interests task), per parent study design.

The total number of resting state runs per participant ranged between 1 and 7, though the median was 1 resting state run. Participants completed between 1 and 3 anatomical scans at each study visit. For analyses, the functional scan with lowest average motion (median framewise displacement) out of any study visit and the highest quality structural scan at that same visit (determined via visual inspection and Computational Anatomy Toolbox (CAT) output) were chosen for analysis. Thus, a total of one structural and one functional run were analyzed per participant.

### 2.3. MRI processing

Neuroimage data storage and processing took place on the Vanderbilt University Institute of Imaging Science Center for Computational Imaging XNAT (Harrigan et al., 2016; Huo et al., 2018). The processing pipelines are available through GitHub (Functional preprocessing: https://github.com/baxpr/connprep/tree/v2.1.0; Connectivity calculation: https://github.com/baxpr/conncalc/tree/v1.1.0).

### 2.4. Structural preprocessing

T1-weighted anatomical images were preprocessed using the CAT12 extension to Statistical Parametric Mapping (SPM, version 12) software (Gaser et al., 2022). Anatomical images were skull stripped and normalized to MNI space. Quality assurance measures at this stage included the overall rated image quality (IQR) value, computed as part of the CAT12 toolbox and representing a quality measure across signal noise and bias. These numbers range from 0 to 100 and are interpreted on a letter grade scale (Gaser et al., 2022). Participants with IQR< 70 were excluded from further analyses (*n* = 2 AUT, *n* = 1 N-AUT). Additionally, original structural images and MNI-normalized structural images were visually examined for quality by two trained raters, resulting in 2 exclusions for artifacts (both N-AUT).

### 2.5. Functional preprocessing

For each functional run, head motion realignment was completed using a two-stage SPM procedure with six head motion parameters. These motion parameters and their first derivates were also included as nuisance regressors in subsequent analyses. Functional images were co-registered to structural images using a rigid body transform and then normalized to MNI space using the previously computed CAT12 transform. Additional steps taken to reduce noise in the fMRI signal include bandpass filtering (between 0.01 and 0.10 Hz), aCompCor, and mean gray matter signal regression (GSR). Though the potential for distance-dependent artifacts is a particular concern when using GSR (Ciric et al., 2017; Gotts et al., 2013), it was ultimately included since in large-scale evaluations across varying leniency of motion exclusion thresholds, GSR was generally found to improve performance across a wide range of quality control metrics. Particularly, GSR reduced the degree of significant connections based on motion levels alone more than was achieved by either aCompCor or ICA-AROMA without GSR (Parkes et al., 2018). To further address the potential for lingering motion-related confounds after the application of these steps, the term of framewise displacement was included as an additional control regressor in the statistical model (see below).

Quality assurance for functional preprocessing included two criteria for determining acceptable levels of motion/noise due to motion. Framewise displacement (FD) and derivative of temporal variance (DVARS) were calculated. Individuals were excluded for either high average motion (FD>0.5 mm, *n* = 3 AUT) or a high number of volumes with extreme motion (FD>1 mm and DVARS>5 %, *n* = 3 AUT) or both (*n* = 3 N-AUT). The quality of co-registration between structural and functional images was also visually examined by two trained raters, resulting in 3 additional exclusions (*n* = 1 AUT, *n* = 2 N-AUT).

### 2.6. Insula subregion definition

Posterior, middle, and anterior insula regions of interest (ROIs) were defined for the left and right hemispheres following protocols used previously in Failla et al. (2017) as traced in adults and based on Farb et al.’s (2013) cytoarchitectonic subdivisions among the insular gyri. Briefly, from Farb et al.’s (2013) 8 subdivisions, the two most posterior regions, the three middle regions, and the three most anterior regions were combined per hemisphere.

### 2.7. Statistical analysis

Whole brain seed-based connectivity was analyzed with each insula ROI (6 total: right and left * PI, MI, and AI), respectively, as a seed. In the first analytic stage, a Z map per individual scan was computed as the correspondence between each voxel and the average signal within each insular seed.

In the second analytic stage, connectivity strength was assessed relative to individual predictors. The individual predictors used as covariates were: age, group, biological sex, motion (median FD), and protocol. The term of protocol was used as a control covariate to capture any variation that may be attributed to acquisition differences between protocols 1 and 2–3 and/or in task spillover effects. Though models with linear (t test for Age) and non-linear (F test for Age+Age^2^) age terms were compared, findings were well-captured by linear terms alone; thus, age-related findings are presented from the results of the linear model. In addition, a model was run in the subset of participants (*n* = 56) that had completed the BPQ Awareness subscale to assess interoceptive self-report. Threshold-free cluster enhancement (TFCE) using *n* = 5000 permutations and, given that type 2 errors were also a risk with our sample size and that our approach was exploratory in nature, a less conservative familywise error rate of p = 0.10 (compared to a standard p = 0.05) was implemented to determine significant clusters per predictor. In addition, see that when the traditional p < 0.05 threshold is applied to an alternative FDR approach, the results are not spatially broader for several key contrasts (Supplemental Figure 1). Lastly, a voxel threshold of k > 5 voxels was implemented to determine the final reported clusters.

## 3. Results

### 3.1. Average patterns: insular connectivity by subregion

**Fig. 3.**
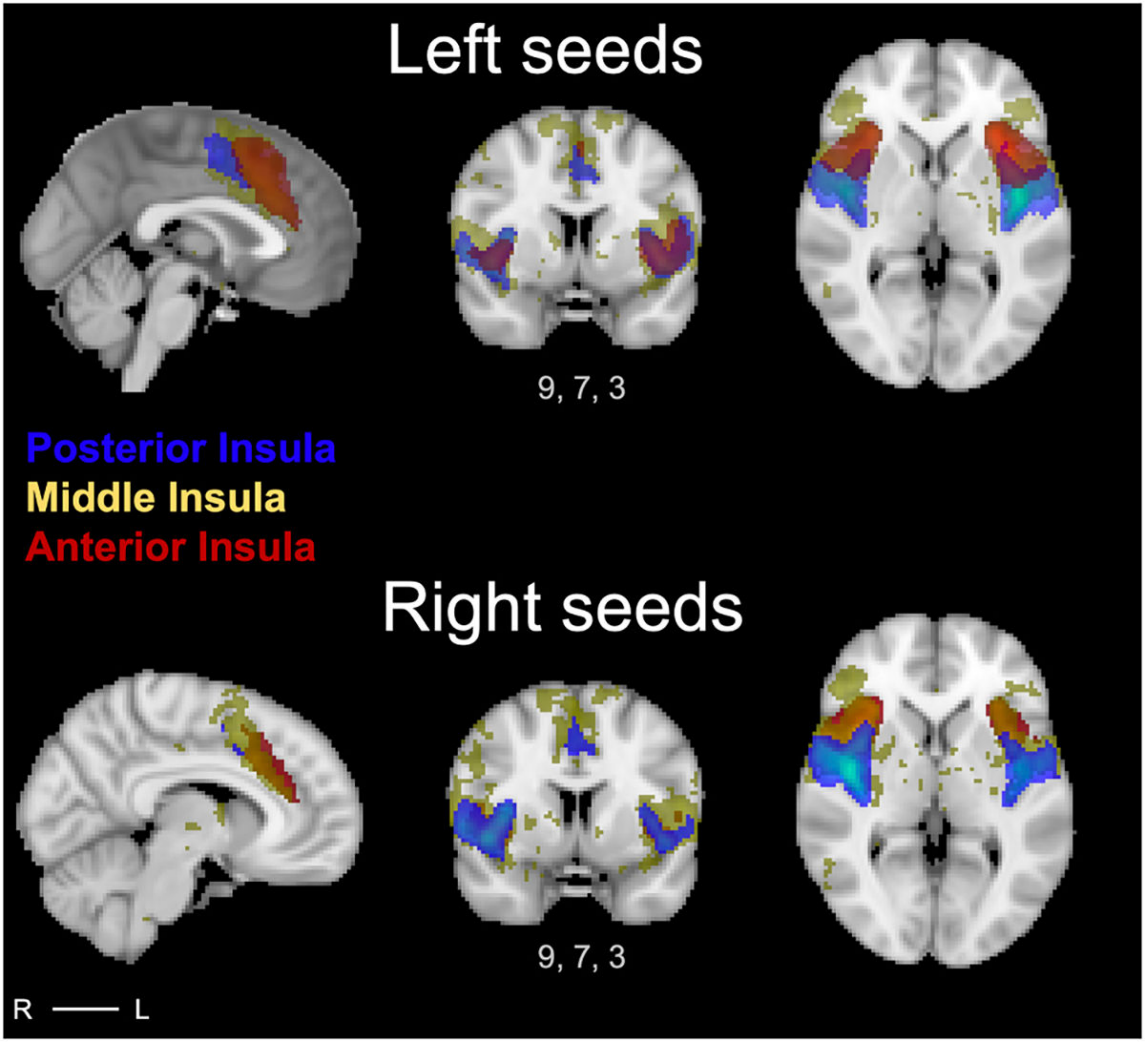
Average connectivity patterns by insular seed. Connectivity maps significantly associated (at Z > 3) with the model intercept term, i.e., the average connectivity patterns of each seed. The top panel shows the left seeds (posterior, middle, and anterior) and the bottom panel shows the right. Regions are colored by seed: blue=posterior, yellow=middle; red=anterior. Significant regions include insular cortex and anterior cingulate. Images shown in radiological convention.

The intercept term of the model (i.e., reflecting average functional connectivity patterns of each seed) is illustrated in Fig. 3. As expected, functional connectivity with each insula subregion seed was strongest within the interoceptive network, including insular cortex and anterior cingulate. The posterior-anterior mapping of the regions of association matched the posterior-anterior mapping of the seeds.

### 3.2. Findings by age: combined sample

**Fig. 4.**
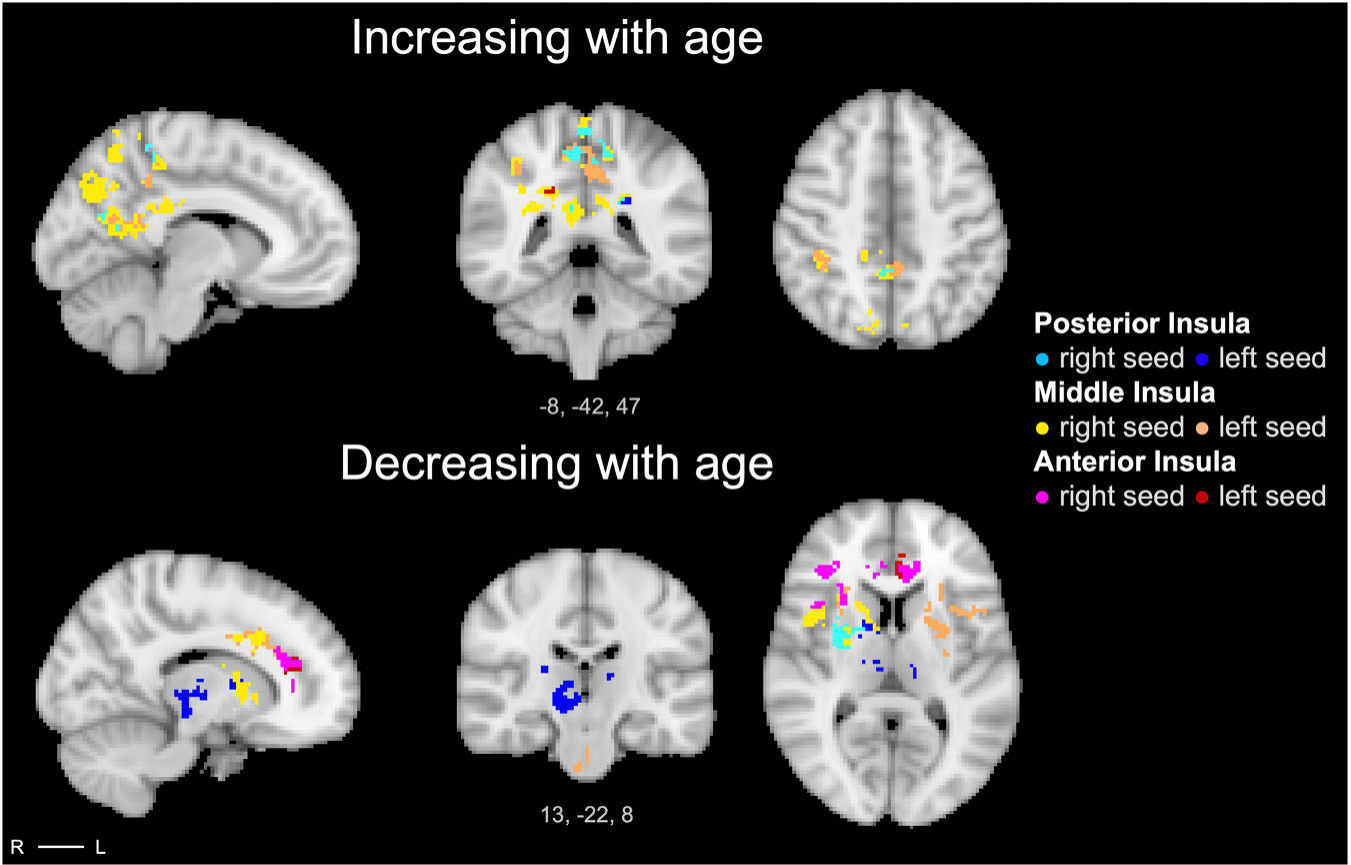
Clusters of significant associations between insula connectivity and linear age term in combined sample, by seed and direction. Significant clusters in which insula functional connectivity increases and decreases with age are shown (using threshold-free cluster enhancement, p_FWE_<0.10). Increasing connectivity with age is shown in areas including precuneus, supplementary motor cortex, and posterior cingulate. Decreasing connectivity with age is shown in areas including the thalamus, insula, and putamen. Clusters are colored by seed (posterior: light/dark blue, middle: yellow/orange, and anterior: red/pink) as shown in figure legend. Images are shown in radiological convention.

The areas of increasing functional connectivity with age were highly overlapping among the insular seeds, though no significant associations were identified for the right AI (Table 2, Fig. 4). The seed regions with the greatest number of clusters were the right PI and right and left MI. Prominent associations were identified with the posterior cingulate (right MI: *k* = 1773 voxels) and the precuneus (right PI: *k* = 209 voxels and left MI: *k* = 219 voxels). Several other findings were localized to white matter, including the sole findings for the left PI (*k* = 14 voxels) and left AI (*k* = 16, 9, 6 voxels, respectively). Most of the other regions identified were also in associative sensory regions spanning both hemispheres, including right supplementary motor cortex, right supracalcarine cortex, right superior parietal lobule, and left lateral occipital cortex.

There were also numerous clusters of significantly decreased associations with age (Table 3, Fig. 4). For the PI, the most prominent areas included the thalamus (left posterior seed: *k* = 292 voxels) and putamen (right posterior seed: *k* = 255 voxels). Many of the other clusters were in the subcortex or surrounding insular areas. For the MI, there were clusters identified in the subcortex, insular/opercular areas, and anterior cingulate. Prominent clusters also encompassed the white matter surrounding these insular/opercular areas (left MI: *k* = 400 voxels, right MI: *k* = 668 voxels). For the AI, most of the clusters of decreasing connectivity with age were in the anterior cingulate, surrounding middle/anterior insula regions, and frontal orbital cortex.

### 3.3. Findings by group

The only seed for which significant group differences were identified was the left PI (Fig. 5). There was one cluster that was associated with increased functional connectivity in the AUT group compared to N-AUT group, within the left lateral occipital cortex (*k* = 62 voxels, MNI_peak_=−12, −62, 58, TFCE=520.57, *p*_*FWE*_=0.017).

### 3.4. Relationships with interoceptive reports

**Fig. 6.**
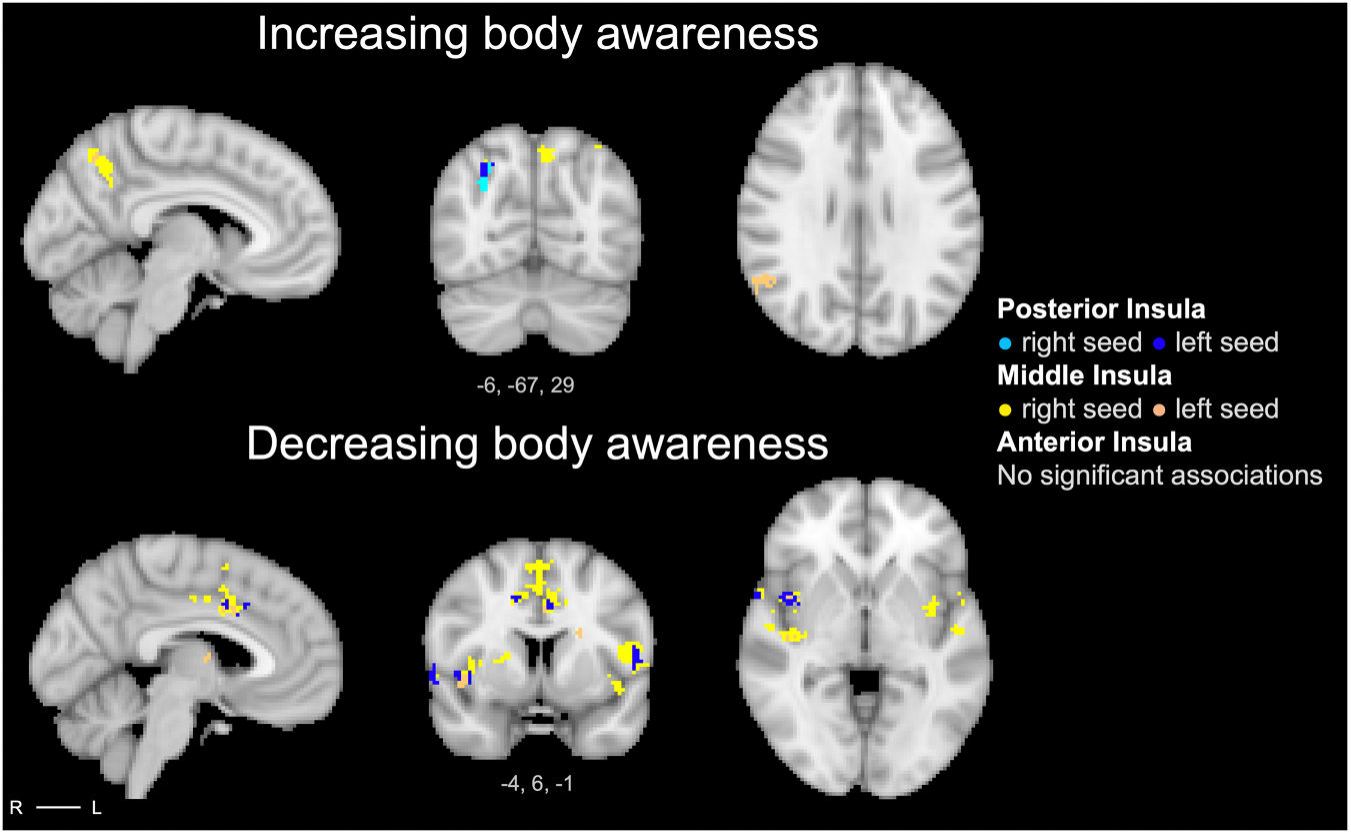
Clusters of association between insula subregions and bodily awareness, by seed. Significant clusters in which insula functional connectivity increases and decreases with body awareness as measured by the BPQ (Porges, 1993) are shown (using threshold-free cluster enhancement, p_FWE_<0.10). Increasing connectivity with BPQ scores is shown in right lateral occipital cortex. Decreasing connectivity with BPQ scores is shown in areas including mid-insula, white matter, anterior cingulate, precentral gyrus, and temporal pole. Clusters are colored by seed (posterior: light/dark blue, middle: yellow/orange, and anterior: red/pink) as shown in figure legend. Images are shown in radiological convention.

Prominent clusters relating to increased BPQ awareness scores overlapped for both the left and right PI seeds, localized to right lateral occipital cortex (Table 4, both peaks at 28, −62, 32; *k* = 78 clusters, *p* = 0.033 left seed and *k* = 35 clusters, *p* = 0.041 right seed; Fig. 6). The right MI seed also showed several similar associations with right and left lateral occipital cortex. A few other clusters were identified with the MI seeds, with areas such as the angular gyrus, precuneus, and superior parietal lobule.

However, the left PI seed and both MI seeds showed many more areas of association with decreased bodily awareness scores (Table 5, Fig. 6). Broadly, these encompassed the primary interoceptive network, including the thalamus, insula, and anterior cingulate. For the left PI seed, the largest of these was in the right MI (*k* = 63, *p* = 0.030). For the MI seed, most prominent clusters were identified in somatosensory cortex in the postcentral gyrus. Other areas identified for the PI and MI included several frontal/prefrontal areas, specifically the precentral gyrus, supplementary motor cortex, and middle frontal gyrus. A few clusters were identified in temporal areas, including the temporal pole and primary auditory cortex. No significant associations were identified for either AI seed, in either direction.

## 4. Discussion

### 4.1. Overview

To summarize, the major findings with age were increasing insular functional connections with parietal, multimodal areas and decreasing functional connections with thalamic, subcortical, and core interoceptive areas. One lateral (occipital/parietal) area was increased in the autistic compared to non-autistic group. In the BPQ subsample, decreased functional connectivity among core interoceptive areas was associated with greater reported awareness. Many of these findings highlight a potential distinction between “greater” processing of interoceptive cues (which could potentially be considered as increased functional connectivity within interoceptive-related regions and/or an overall increased number of significant functional connections) and what may be considered adaptive interoception within a developmental framework. Given this distinction, these findings align well with predictive coding frameworks of interoception and suggest several paths forward for how we understand interoceptive development and potential differences or difficulties in autism.

### 4.2. General development of insula connectivity

#### Decreasing connectivity with age:

Age-related functional connectivity patterns combined across groups revealed a few common themes. There were more clusters of decreasing than increasing insula connectivity with age, reflecting widespread cortical pruning that is typical of adolescent and adult brains (Hua & Smith, 2004; Innocenti & Price, 2005; Katz & Shatz, 1996). Prominent areas of decreases were generally other insula subregions or sub-cortical regions, including thalamus, striatum, and brainstem. This suggests that, over time, primary interoceptive perception becomes a less direct representation of bodily signals as conveyed in these subcortical regions, consistent with a developmental perspective on interoceptive learning (Ainley et al., 2016; Barrett & Simmons, 2015). More surprisingly, the MI and AI showed patterns of decreasing connectivity with age in “higher order” areas in the broader interoceptive system such as the anterior cingulate and frontal opercular, orbital, and frontal gyri. Combined, these changes may reflect adaptive learning wherein signals are increasingly accurately predicted over time and thus, less conscious effort is required to process and respond to bodily needs. Since interoceptive cues are more able to be fully predicted than exteroceptive sensory systems (Barrett & Simmons, 2015), this may be one way in which the interoceptive sensory system diverges from other systems. Less effortful monitoring of these signals may be enabled via the increasing efficiency in cardiovagal autonomic control from childhood to early adulthood (Lenard et al., 2004). Further support for this interpretation comes from interoception task findings (Failla et al., 2020). Failla et al. (2020) found that, compared to children, adults showed greater increases in insula response to heartbeat counting but that this increase did not correspond to better task performance (though noting that Mash et al. (2017) found interactions between group and IQ such that age-related improvements may occur specifically in non-autistic individuals with IQ scores < 115). Together, these findings suggest that, by adulthood, significant neural resources are only engaged in cardioception during conditions of explicit attention (i.e., during a heartbeat counting experiment or when heart rate is elevated).

#### Increasing connectivity with age:

Common patterns of increasing insular connectivity with age can also be interpreted as reflecting increasing efficiency of interoceptive prediction. All three insular subdivisions showed increasing connectivity with posterior parietal cortex, covering broad, overlapping multimodal areas, including white matter regions that may conceivably link these areas (Gore et al., 2019). This is in line with expectations for increasing integration of interoceptive signals with multimodal sensory input, action planning, and an abstracted sense of self identity (Palmer & Tsakiris, 2018).

### 4.3. Autistic differences in PI connectivity

Group differences in insular connectivity were specific to the left PI seed, identifying one main cluster in which the autistic group showed heightened connectivity compared to the non-autistic group, localized to left lateral occipital cortex (LOC) per our chosen atlas, though our identified area may be more parietal than traditional definitions of LOC (Grill-Spector et al., 2001; Nagy et al., 2012). The direction of this finding contrasts prior adolescent samples (Ebisch et al., 2011; Francis et al., 2019) and may be influenced by having a sample that includes older adults. A potential confound to consider is the relative age of our two groups, since the non-autistic group had a somewhat higher age than the autistic group. However, since this cluster occurred in an area closer to those that were increasing with age, yet is elevated in the younger autistic sample, the age differences between our groups do not seem to the sole explanation for these findings.

The identified heightened cluster corresponds to functional areas related to mental imagery, especially processing speed and time estimation (Forn et al., 2013; Sack et al., 2002). Craig (2009)asserts the essential role of the insula in the subjective perception of time, as linked to the cardiac cycle. Thus, increased connectivity between the posterior insula and this lateral area might suggest that temporal aspects of self-referential processing may be especially anchored to interoceptive information in autistic individuals. Otherwise, the null findings by group across other insular seeds call into question whether findings of reduced interoceptive accuracy, as suggested by aggregate findings of reduced heartbeat counting accuracy in autism (Williams et al., 2022) might have a nuanced neural explanation that extends beyond reduced perception or attention towards these signals.

### 4.4. Relationship with bodily awareness

We identified several regions in which PI and MI connectivity covaried with self-reported bodily awareness. Coinciding across multiple of these seeds, trends with increased bodily awareness were found with lateral parietal/occipital cortex clusters. These areas are near, though not identical to, areas identified in the age findings, which may additionally relate to self-referential processing (e.g., Forn et al., 2013; Sack et al., 2002). Enhanced connectivity between insula and lateral occipital/parietal cortices in individuals with higher bodily awareness may suggest that individuals who more closely relate interoception with their sense of self perceive these cues more readily.

Conversely, areas related to reduced body awareness were identified in key interoceptive processing regions, including within insular cortex and anterior cingulate. These further included white matter regions, perhaps related to recent findings supporting the role of glial cells in efficient interoceptive processing (Fabbri et al., 2023). However, the negative relationship between reported bodily awareness and functional connectivity within the interoceptive network is initially surprising, since an intuitive expectation might be that stronger within-network communication leads to greater awareness of these signals. Kleckner et al. (2017) previously found that within-interoceptive network connectivity related positively to individuals’ attunement (measured as reported stress levels) to their stress response (measured by galvanic skin response), providing one basis to expect a positive relationship. However, in our sample, this may reflect an overly modular network that makes it difficult to broadly contextualize interoceptive signals.

Since the origin of data collection for this study, the literature has greatly expanded in the number of published interoception questionnaires and the understanding of the multifaceted aspects of interoception that may be captured by different types and wording of questions (Trevisan et al., 2023; Trevisan et al., 2021). The BPQ Awareness subscale reflects the perceived strength and frequency of a broad number of interoceptive sensations, without a focus on the interpretation or context of these sensations and may reflect a less adaptive form of interoceptive awareness. Therefore, these findings as compared to different measures of interoceptive behaviors might support predictive coding models (Barrett & Simmons, 2015). Across the broad periods of time covered by the BPQ, individuals with strong within-interoceptive network function may predict and meet their needs readily, whereas individuals who predict these signals less readily may remember more of them to report. Further, individuals who relate these signals via parietal cortex connections to a broader sense of self may report greater everyday awareness of these signals. The other aspect of the BPQ Awareness subscale that may be reflected in these parietal cortex connections is that it includes some items that reflect interoceptive sensations that overlap between the viscera and somatosensation, such as feeling bloated (Boccia et al., 2023; Stern et al., 2017). Further study of how functional connections relate to the different, multidimensional aspects of interoceptive experiences and perception across the body boundary is important, including via scales that differentiate parasympathetic versus sympathetic nervous system processing (Porges, 1993), new measures that more clearly define problems and challenges (Fiene et al., 2018), as well as refined measures for use in autistic individuals (Suzman et al., 2021).

Unexpectedly, we did not find any areas of significant associations between AI seeds and BPQ Awareness scores. This may reflect the nuances described above for the BPQ’s focus on direct interoceptive sensation, relative to the more abstracted role of emotional awareness served by the AI. A related possibility is that higher inter-individual variability in the more widely-connected AI relative to PI and MI may have limited our ability to detect significant relations in our smaller subsample with this measure. Thus, these findings build a foundation for understanding how neural communication within interoceptive regions leads to bodily awareness, but further work is needed to understand when these patterns may become disruptive to the individual’s functioning.

### 4.5. Structure-function relationship

One caveat in interpreting the results of this study is that we solely focused on functional connectivity and thus findings may be complex and nuanced in how they relate to the underlying structural pathways and neuronal activity. However, there are several interesting parallels to consider with the structural literature. This study provides potential interoceptive developmental information, which may align both with known interoceptive structural patterns and the important implications of diverging patterns in autism. There is work to suggest decreased alignment between structural and functional network development in the insula, largely driven by functional differences, further relates to the degree of autism-associated behaviors as measured using the ADOS (Dong et al., 2023). Insular surface area networks were also found to be more segregated in autistic vs. non-autistic individuals, a finding which was relatively stable with age (Zheng et al., 2021). Lastly, one large structural study suggests that patterns of PI cortical thickness diverge with age such that in older autistic individuals, PI cortical thickness is particularly reduced compared to non-autistic individuals (Zoltowski et al., 2021). These changes in brain structure with age may possibly contribute to differences in functional connections identified here, and perhaps even be exaggerated in older autistic compared to younger autistic individuals.

### 4.6. Implications for health and well-being

Interoceptive-related interventions, including explicitly targeting cardiac perception (Quadt et al., 2021) as well as mindfulness approaches that show close targeting of interoceptive systems (Hatfield et al., 2023), have been shown to be helpful for ameliorating anxiety in autistic samples. However, these studies are early in their exploration, and a better understanding of the functional development of interoceptive systems informs the best targets for these interventions. Changes with age in the relative degree to which interoceptive cues are integrated with environmental information suggest that an ideal intervention for an adult with interoceptive difficulties may differ from that for a child with similarly presenting difficulties, in ways that may extend beyond cognitive and linguistic development. Further, the finding of one heightened insula-occipital/parietal connection in the autism group, if confirmed through replication, lends further support for the promise of mindfulness interventions in autism, if such internal-external integration has a strong neural basis.

The BPQ findings also provide some ideas to personalize intervention targets for heterogeneous individuals. One other possibility to consider for the limited group differences we identified may lie in the broad range of BPQ scores in the autistic compared to non-autistic group. Autistic participants showed a wider range of scores at both ends of the scale, including reduced and enhanced awareness. Thus, autistic heterogeneity may reflect distinct subgroups representing deviations from healthy interoceptive processing in different directions. This is a promising area for future studies to explore and incorporate into precision medicine approaches.

### 4.7. Limitations and future directions

Though our sample size is greater than prior studies of PI and MI connectivity by age (Ebisch et al., 2011; Francis et al., 2019), this work would still benefit from larger-scale studies, especially to understand differential trends in autistic versus non-autistic development. Normative modeling approaches in larger samples would provide a meaningful extension of this work, to understand more precise developmental trends and potential trajectory differences in autism. Further, our findings by age have the potential to be influenced by differences in image acquisition and possible task carry-over effects between our three protocols. Though each of our three protocols did include youth and adult participants across a broad age range, these distributions were not fully matched between samples. Thus, further work is needed to disentangle differences in resting versus active interoceptive network development. Future studies may also implement systems-level approaches that characterize interoceptive development relative to reliably identified resting state networks, such as a recently available quantitative network toolbox (doi: 10.1101/2024.06.17.599426).

The major patterns from our findings are also important to replicate in future work. Since our p-value threshold was more liberal than commonly used, some of our findings may indeed be false positives. Another technical limitation to consider is that, though there has been steady progress in correcting for movement-related artifact in resting state fMRI, findings in functional connectivity are influenced by choice of preprocessing pipeline and it remains difficult to determine which best reflects true differences (He et al., 2020; Parkes et al., 2018). This provides a caveat to the reliability of our identified heightened functional connection between posterior insula and left lateral occipital cortex in the autistic group; thus, these findings are presented as potentially interesting to consider relative to the behavioral interoception literature in autism, but far from conclusive about brain processing differences in autism. The length of the resting state scans is also a factor to consider in regards to the reliability of these findings, since we completed approximately 6 minute scans, whereas reliability of resting state results continues to improve through 12 minutes of scanning (Birn et al., 2013).

There are also several limitations to consider specific to interpreting the BPQ findings. Our sample size for these analyses was much smaller than ideal to address brain-behavior correlations (Marek et al., 2022), which remains a challenge in many such studies. This challenge especially limits us in understanding autistic heterogeneity. As mentioned, autistic individuals reported a wide range of bodily awareness levels, with individuals at both the highest and lowest ends of the scale. Thus, one useful approach to better understand the link between interoceptive brain systems and interoceptive experiences might be to subgroup individuals based on these higher and lower awareness patterns, in samples that are large enough to do so. There are other heterogeneous individual factors that may influence interoceptive processing that may be more closely explored than what we examined here, such as language, IQ, the interaction with other domains of autism (such as social features, motor behaviors, and other sensory processing domains). Lastly, the understanding of interoceptive processing and its development may be refined through delineating specific sub-modalities, e.g. by using measures that distinguish cardiac versus respiratory processing, etc.

## 5. Conclusions

These findings extend the existing literature of how functional connectivity of insular cortex changes over development, across the posterior, middle, and anterior subdivisions. In general, our findings support theories for how our expectations for these signals become increasingly well predicted and integrated with multimodal cortices over the course of development. This shift may further relate to changes in bodily awareness, including the ability to adaptively tune out certain interoceptive sensations. Minimal differences were identified between the autistic and non-autistic groups; those that were found were localized to the left posterior insula seed and may specifically relate to experienced perception of time. Thus, these findings identify tentative differences in how autistic individuals may integrate and contextualize their interoceptive sensations, though work in larger samples is needed to confirm these patterns and understand their link to specific developmental stages.

## Supplementary Material

1

## Figures and Tables

**>Fig. 5.**
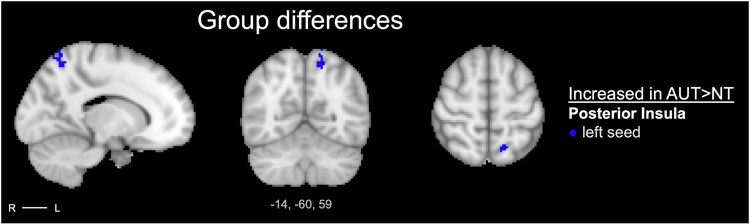
Clusters of group-specific associations with posterior insula connectivity. Differences in posterior insula functional connectivity by group were limited to one cluster, between left posterior insula and left lateral occipital cortex, shown here in blue. Functional connectivity with this region was increased in the autistic compared to non-autistic group (using threshold-free cluster enhancement, pFWE<0.10). Images are shown in radiological convention.

**Table 1:** Participant characteristics by diagnostic group.

Group	*n*	Age (years)	Sex (% F)	Race	Motion		*n* with BPQ	BPQ Aware
					MdnFD	DVARS		

N-AUT	71	20.2 (7–53)	28.2 %	1.4 % American Indian / Alaska Native 4.2 % Asian 8.5 % Black or African American 0 % Multiracial 76.0 % White or Caucasian	0.185 (0.08–0.36)	1.99 (1.19–2.94)	24	2.87 (1.77–3.46)
AUT	59	16.9 (7–54)	28.8 %	1.7 % American Indian / Alaska Native 1.7 % Asian 3.4 % Black or African American 6.8 % Multiracial 72.9 % White or Caucasian	0.196 (0.09–0.37)	1.92 (1.46–2.54)	32	2.75 (1.12–4.42)
*p*		0.06	> 0.99	0.14	0.36	0.23		0.49

Characteristics per group are summarized as mean (range) for continuous variables and percentage (%) for categorical variables. Race was self-reported by study participants; no participants self-reported their race as Native Hawaiian or Pacific Islander. P-values of tests for differences in group characteristics are reported from two-sample t-tests for continuous variables and chi-square tests for categorical variables. No participant characteristics significantly differed between the groups, though the autistic participants were somewhat younger on average. Abbreviations: N-AUT: non-autistic, AUT: autistic, n: included sample size, F: female, MdnFD: median framewise displacement (millimeters), DVARS: derivative of temporal variance (arbitrary units), BPQ: Body Perception Questionnaire, Awareness subscale (Porges, 1993)

**Table 2:** Summary of significant clusters for increasing age term by seed.

Insula subregion	Seed hemisphere	#	Location	Coordinates	TFCE	p_FWE_

Posterior	L	14	White matter (L)	−24, −44, 26	464.34	0.057
Posterior	R	209	Precuneus (R)	4, −44, 50	491.62	0.036
Posterior	R	39	Precuneus (L)	−16, −54, 18	485.69	0.04
Posterior	R	36	Precuneus (L)	−14, −64, 18	449.73	0.07
Posterior	R	20	Precuneus (L)	−4, −72, 34	447.2	0.073
Posterior	R	19	Precuneus; Lingual gyrus	0, −54, 8	453.63	0.066
Posterior	R	19	Supplementary motor cortex (R)	2, −10, 72	465.92	0.054
Posterior	R	6	Posterior cingulate gyrus (R)	10, −46, 22	434.66	0.088
Posterior	R	5	Precuneus (L)	−10, −78, 38	436.37	0.086
Posterior	R	5	Precuneus (L)	−4, −44, 48	430.91	0.093
Posterior	R	5	White matter (L)	−24, −42, 26	434.54	0.088
Posterior	R	4	White matter (L)	−22, −48, 26	435.64	0.087
Middle	L	219	Precuneus cortex (L)	−4, −44, 46	575.81	0.007
Middle	L	74	White matter/precuneus cortex (L)	−10, −56, 18	472.43	0.042
Middle	L	45	Superior parietal lobule (R)	40, −36, 46	499.95	0.026
Middle	L	7	Precuneus cortex (R)	4, −52, 64	429.16	0.088
Middle	L	5	Precuneus cortex (L)	−10, −68, 22	438.32	0.075
Middle	L	5	Precuneus cortex (R)	4, −44, 66	428.13	0.089
Middle	R	1773	White matter/posterior cingulate (R)	8, −44, 18	753.05	0
Middle	R	341	Precuneus (R)	2, −44, 46	531.66	0.019
Middle	R	194	Lateral occipital cortex (L)	−12, −60, 60	523.63	0.021
Middle	R	129	Precuneus (R)	2, −44, 70	485.79	0.039
Middle	R	17	Superior parietal lobule (R)	34, −56, 62	449.98	0.069
Middle	R	10	White matter (L)	−18, −52, 30	437.72	0.083
Anterior	L	16	White matter (R)	16, −36, 30	453.8	0.065
Anterior	L	9	White matter/precuneus (R)	22, −48, 20	468.37	0.052
Anterior	L	6	White matter (R)	18, −46, 26	439.43	0.081

Model: **Age** + Group + Biological Sex + Motion + Protocol. Entries are grouped by seed and then ordered by number of voxels (#, largest to smallest). Locations were assigned as the top match using the Harvard-Oxford Cortical and Subcortical Atlases. If a second match differed by less than 5 % probability, this area was included as well. Other columns include, for the peak cluster, the corresponding MNI coordinates, threshold-free cluster enhancement score, and family-wise error p-value (p_FWE_).

**Table 3:** Summary of significant clusters for decreasing age term by seed.

Insula subregion	Seed hemisphere	#	Location	Coordinates	TFCE	p_FWE_

Posterior	L	292	Thalamus (R)	8, −18, 2	536.69	0.016
Posterior	L	67	Insula (Anterior, R)	30, 10, 14	486.53	0.036
Posterior	L	39	Caudate (R)	14, 4, 8	450.02	0.065
Posterior	L	36	Frontal operculum cortex (R)	42, 10, 12	470.62	0.047
Posterior	L	20	Putamen (R)	26, −2, 4	440.24	0.076
Posterior	L	19	Insula (Mid-Anterior, L)	−32, 14, 6	453.26	0.062
Posterior	L	19	Thalamus (R)	18, −10, 12	450.68	0.064
Posterior	L	14	Thalamus (L)	−12, −22, 10	435.29	0.082
Posterior	L	10	Thalamus (L)	−18, −14, 18	433.54	0.084
Posterior	L	10	Insula (Mid, R)	38, 4, 4	432.35	0.086
Posterior	L	9	Putamen (L)	−24, 4, 8	435.11	0.082
Posterior	L	5	Inferior frontal gyrus, p. oper. (R)	46, 14, 14	427.63	0.093
Posterior	R	255	Putamen (R)	26, −6, 8	578.57	0.01
Posterior	R	45	Occipital pole (V1, L)	−8, −96, −2	469.13	0.052
Middle	L	643	White matter/paracingulate (R)	14, 18, 34	651.89	0.003
Middle	L	483	Putamen (L)	−28, 2, 6	538.41	0.018
Middle	L	400	Frontal operculum (R)	42, 10, 12	585.50	0.009
Middle	L	54	Anterior cingulate (R)	8, 26, 16	474.86	0.048
Middle	L	29	White matter (L)	−18, −12, 18	463.84	0.057
Middle	L	26	White matter (L)	−4, −10, 22	471.35	0.051
Middle	L	21	White matter/precentral gyrus (R)	34, −14, 56	454.79	0.065
Middle	L	17	Brain stem	0, −20, −40	458.12	0.062
Middle	L	14	Paracingulate (R)	10, 8, 48	448.44	0.072
Middle	L	12	White matter/pallidum (R)	12, 4, 0	441.50	0.080
Middle	L	11	White matter (L)	−10, 4, −4	462.05	0.059
Middle	L	11	Brain stem	4, −24, −38	445.92	0.075
Middle	L	10	Central operculum (L)	−46, −4, 10	439.64	0.082
Middle	L	9	Putamen (L)	−28, −12, 8	434.64	0.088
Middle	L	7	Brain stem	10, −42, −30	456.91	0.063
Middle	L	7	White matter (L)	−26, −16, 20	435.15	0.088
Middle	L	7	Superior frontal gyrus (R)	12, 16, 48	433.85	0.089
Middle	L	7	Central operculum (L)	−38, 6, 16	433.00	0.090
Middle	L	6	Brain stem	0, −26, −26	457.31	0.063
Middle	L	6	Brain stem	−6, −26, −38	439.93	0.082
Middle	L	5	White matter (L)	−12, 8, 22	436.24	0.086
Middle	L	5	Brain stem	2, −30, −30	429.72	0.095
Middle	R	668	Frontal operculum (R)	42, 10, 12	675.63	0.002
Middle	R	65	Temporal pole (R)	44, 12, −16	482.77	0.043
Middle	R	34	White matter/paracingulate (R)	14, 18, 34	479.02	0.046
Middle	R	23	Insula (Posterior, R)	38, −10, −6	453.52	0.067
Middle	R	21	White matter (L)	−18, −12, 18	459.21	0.062
Middle	R	15	White matter (L)	−30, 6, 14	450.55	0.07
Middle	R	14	Anterior cingulate (R)	6, 20, 28	440.06	0.082
Middle	R	11	Paracingulate (L)	−14, 18, 30	459.99	0.061
Middle	R	11	Anterior cingulate (R)	14, 6, 34	447.94	0.073
Middle	R	7	White matter (R)	12, −2, 34	439.33	0.083
Middle	R	6	White matter/precentral gyrus (R)	50, 2, 12	445.63	0.076
Middle	R	5	Anterior cingulate (L)	−14, 30, 20	442.9	0.079
Middle	R	5	Anterior cingulate (L)	−6, 14, 32	436.68	0.086
Middle	R	5	Putamen (L)	−24, 6, 0	436.45	0.087
Middle	R	5	Anterior cingulate (R)	6, 32, 18	432.84	0.091
Anterior	L	345	Anterior cingulate (R)	14, 32, 22	574.81	0.013
Anterior	L	33	Insula (Anterior, R)	32, 18, −4	461.99	0.061
Anterior	L	28	Insula (Anterior, L)	−28, 24, −4	469.1	0.055
Anterior	L	15	Insula (Mid-Anterior, L)	−30, 14, −6	467.2	0.057
Anterior	L	12	Paracingulate (L)	−4, 36, 28	454.18	0.068
Anterior	R	474	Insula (Mid-Anterior, R)	32, 16, −4	685.96	0.002
Anterior	R	364	Anterior cingulate (R)	14, 32, 22	625.15	0.005
Anterior	R	71	White matter/frontal pole (R)	36, 34, 8	508.27	0.029
Anterior	R	67	Frontal orbital cortex (L)	−28, 6, −16	520.5	0.024
Anterior	R	31	Frontal operculum (R)	40, 16, 6	466.64	0.055
Anterior	R	11	Anterior cingulate (L)	−10, 30, 24	452.58	0.068
Anterior	R	9	Anterior cingulate (L)	−14, 34, 18	447.2	0.074
Anterior	R	7	Insula (Anterior, L)	−28, 26, 2	434.2	0.089

Model: **Age** + Group + Biological Sex + Motion + Protocol. Entries are grouped by seed and then ordered by number of voxels (#, largest to smallest). Locations were assigned as the top match using the Harvard-Oxford Cortical and Subcortical Atlases. If a second match differed by less than 5 % probability, this area was included as well. Other columns include, for the peak cluster, the corresponding MNI coordinates, threshold-free cluster enhancement score, and family-wise error p-value (p_FWE_).

**Table 4:** Summary of significant clusters by seed for increasing body awareness.

Insula Subregion	Seed hemisphere	#	Location	Coordinates	TFCE	p_FWE_

Posterior	L	78	Lateral occipital cortex (R)	28, −62, 32	600.84	0.033
Posterior	R	35	Lateral occipital cortex (R)	28, −62, 32	516.62	0.041
Posterior	R	5	Lateral occipital cortex (R)	26, −66, 44	462.34	0.089
Middle	L	59	Angular gyrus (R)	58, −56, 28	557.58	0.032
Middle	L	14	Precuneus (L)	−8, −66, 48	499.51	0.066
Middle	L	8	Precuneus (R)	6, −62, 32	484.93	0.079
Middle	R	158	Precuneus (L)	−6, −58, 42	593.17	0.021
Middle	R	60	Superior Parietal Lobule (L)	−32, −54, 52	547.3	0.039
Middle	R	80	Posterior cingulate (L)	14, −42, 34	520.33	0.055
Middle	R	26	Lateral occipital cortex (R)	26, −70, 42	519.58	0.055
Middle	R	7	Lateral occipital cortex (L)	−34, −68, 56	486.83	0.083
Middle	R	8	Superior Parietal Lobule (L)	−26, −48, 42	485.88	0.084
Middle	R	5	Lateral occipital cortex (L)	−36, −60, 56	478.57	0.092
Middle	R	6	Lateral occipital cortex (L)	−32, −62, 48	477	0.094

Model: Age + Group + Biological Sex + Motion + Protocol + **BPQ Awareness**. Locations were assigned as the top match using the Harvard-Oxford Cortical and Subcortical Atlases. If a second match differed by less than 5 % probability, this area was included as well. Other columns include, for the peak cluster, the corresponding MNI coordinates, threshold-free cluster enhancement score, and family-wise error p-value (p_FWE_).

**Table 5:** Summary of significant clusters by seed for decreasing body awareness.

Insula subregion	Seed hemisphere	#	Location	Coordinates	TFCE	p_FWE_

Posterior	L	63	Insula (Mid, Right)	42, 4, −4	596.24	0.03
Posterior	L	28	White matter (R)	22, −24, 30	557.44	0.053
Posterior	L	25	Paracingulate gyrus (L)	−6, 18, 38	537.1	0.071
Posterior	L	25	Precentral gyrus (L)	−54, 6, 8	554.79	0.055
Posterior	L	24	White matter (L)	−16, 2, 34	551.77	0.058
Posterior	L	18	White matter (R)	22, −10, 36	556.55	0.054
Posterior	L	17	Supplementary motor cortex; Anterior cingulate (R)	14, 6, 40	554.86	0.055
Posterior	L	16	Temporal pole (R)	60, 6, −2	533.14	0.076
Posterior	L	11	Frontal operculum, temporal pole (R)	48, 16, −6	521.25	0.09
Posterior	L	10	Precentral gyrus (L)	−30, −10, 42	536.68	0.072
Posterior	L	10	Temporal pole (R)	50, 14, −16	544.21	0.064
Posterior	L	7	Anterior cingulate (R)	6, 12, 36	526.09	0.084
Middle	L	48	Postcentral gyrus (L)	−44, −34, 64	519.78	0.046
Middle	L	32	White matter (L)	−22, 8, 22	503.67	0.058
Middle	L	19	Ventricle/Caudate (L)	−8, 0, 12	485.67	0.074
Middle	L	18	Thalamus (R)	6, −2, 10	491.65	0.068
Middle	L	17	Anterior cingulate (L)	−2, 4, 36	485.14	0.074
Middle	L	16	White matter (L)	−38, −12, 34	481.73	0.078
Middle	L	15	Insula (Mid, Right)	42, 6, −4	491.28	0.068
Middle	L	15	Precentral gyrus (L)	−34, −6, 64	499.36	0.061
Middle	L	9	Planum polare (R)	42, −16, −4	483.32	0.076
Middle	L	7	Superior Parietal Lobule (L)	−26, −44, 72	502.92	0.058
Middle	L	7	White matter (R)	26, −28, 16	479.18	0.081
Middle	L	6	White matter (R)	8, 14, 22	476.8	0.083
Middle	L	5	White matter (midline)	0, −8, 18	472.34	0.088
Middle	R	686	Supramarginal gyrus (R)	52, −28, 32	612.6	0.018
Middle	R	619	Postcentral gyrus (L)	−56, −16, 22	679.91	0.008
Middle	R	429	Postcentral gyrus (L)	−28, −40, 74	597.49	0.022
Middle	R	384	Anterior cingulate (L)	−4, 4, 36	599.03	0.022
Middle	R	201	Thalamus (R)	10, −2, 8	541.49	0.044
Middle	R	188	Insula (Post/Mid, L)/White matter	−36, 0, −6	611.84	0.018
Middle	R	177	Precentral gyrus (L)	−36, −8, 66	552.44	0.038
Middle	R	169	Inferior frontal gyrus, pars opercularis (L)	−46, 6, 12	634.26	0.014
Middle	R	166	Insula (Mid, Right)	42, 6, −4	677.54	0.008
Middle	R	97	Temporal pole (R)	58, 14, −8	508.21	0.065
Middle	R	63	White matter (L)	−28, −24, 12	531.47	0.049
Middle	R	45	Central opercular cortex (S2, L)	−40, −14, 20	513.73	0.061
Middle	R	42	Anterior cingulate (L)	−8, −10, 40	524.54	0.054
Middle	R	33	Superior frontal gyrus (L)	−10, −4, 66	497.38	0.074
Middle	R	29	White matter/Superior temporal gyrus, anterior (L)	−50, −12, −4	549.43	0.04
Middle	R	24	Superior temporal gyrus, anterior (L)	−62, −6, 4	513.58	0.061
Middle	R	21	Supramarginal gyrus (L)	−62, −28, 22	555.52	0.037
Middle	R	19	Postcentral gyrus (R)	48, −14, 30	496.3	0.075
Middle	R	14	Precentral gyrus (R)	26, −8, 68	494.89	0.076
Middle	R	11	Central opercular cortex (S2)/Insula (Mid, L)	−40, −2, 14	499.09	0.073
Middle	R	9	Precentral gyrus (R)	56, 0, 50	532.33	0.049
Middle	R	8	Thalamus (R)	16, −20, 8	499.47	0.072
Middle	R	7	Superior temporal gyrus, anterior (L)	−62, −12, 0	503.23	0.069

Model: Age + Group + Biological Sex + Motion + Protocol + **BPQ Awareness**. Locations were assigned as the top match using the Harvard-Oxford Cortical and Subcortical Atlases. If a second match differed by less than 5 % probability, this area was included as well. Other columns include, for the peak cluster, the corresponding MNI coordinates, threshold-free cluster enhancement score, and family-wise error p-value (p_FWE_). Figure Captions

## Data Availability

Data will be made available on request.

## Appendix A. Supporting information

Supplementary data associated with this article can be found in the online version at doi:10.1016/j.biopsycho.2025.109043.

## Appendix B. Supporting information

Supplementary data associated with this article can be found in the online version at doi:10.1016/j.biopsycho.2025.109043.
