# Supplementary material for: Insular functional connectivity in autistic and non-autistic development
Source: Biol Psychol. Author manuscript; Available in PMC 2026 Jul 1. (PMC13322401; doi:10.1016/j.biopsycho.2025.109043)
Supplement: 1 [file NIHMS2175201-supplement-1.docx]

Insular functional connectivity in autistic and non-autistic development

Alisa R. Zoltowski^a,b,c^, Michelle D. Failla^d^, Fiona Wu^e^, Caitlin A. Convery^b^, Brianna Lewis^b,h^, Neil D. Woodward^b^, Baxter P. Rogers^f,g^, Carissa J. Cascio^a,b,c,h^

SUPPLEMENTAL INFORMATION

Across all cohorts, each participant was asked by MRI technicians during the study screening phase (i.e., prior to scans being scheduled) whether any of the following criteria was applicable to him/her. Endorsing any of the following conditions without a previous successful MRI scan would cause the MRI technicians to cancel the scan.

Possible conditions:

- Alcohol or substance abuse
- Injury by metallic object or foreign body
- History of asthma, allergic reaction, respiratory disease, or reaction to a contrast medium or dye used for an MRI, CT, or X-ray examination
- Anemia or any disease(s) that affects your blood, a history of renal (kidney) disease, renal (kidney) failure, renal (kidney) transplant, high blood pressure (hypertension), liver (hepatic) disease, a history of diabetes, or seizures
- Antipsychotics, mood stabilizers, or noradrenergic-acting medications
- Aneurysm clip(s)
- Cardiac pacemaker
- Implanted cardioverter defibrillator (ICD)
- Electronic implant or device
- Magnetically-activated implant or device
- Neurostimulation system
- Spinal cord stimulator
- Internal electrodes or wires
- Bone growth/bone fusion stimulator
- Cochlear, otologic, or other ear implant
- Insulin or other infusion pump
- Implanted drug infusion device
- Any type of prosthesis (eye, penile, etc.)
- Heart valve prosthesis
- Eyelid spring or wire
- Artificial or prosthetic limb
- Metallic stent, filter, or coil
- Shunt (spinal or intraventricular)
- Vascular access port and/or catheter
- Radiation seeds or implants
- Swan-Ganz or thermodilution catheter
- Medication patch (Nicotine, Nitroglycerine)
- Any metallic fragment or foreign body
- Wire mesh implant
- Tissue expander (e.g., breast)
- Surgical staples, clips, or metallic sutures
- Joint replacement (hip, knee, etc.)
- Bone/joint pin, screw, nail, wire, plate, etc.
- IUD, diaphragm, or pessary
- Dentures or partial plates
- Tattoo or permanent makeup above the waist
- Body piercing jewelry (that cannot be removed)
- Hearing aid (Remove before entering the room)
- Other implant
- Breathing problem or motion disorder
- Claustrophobia
- Prior problems during MRI or CT procedure


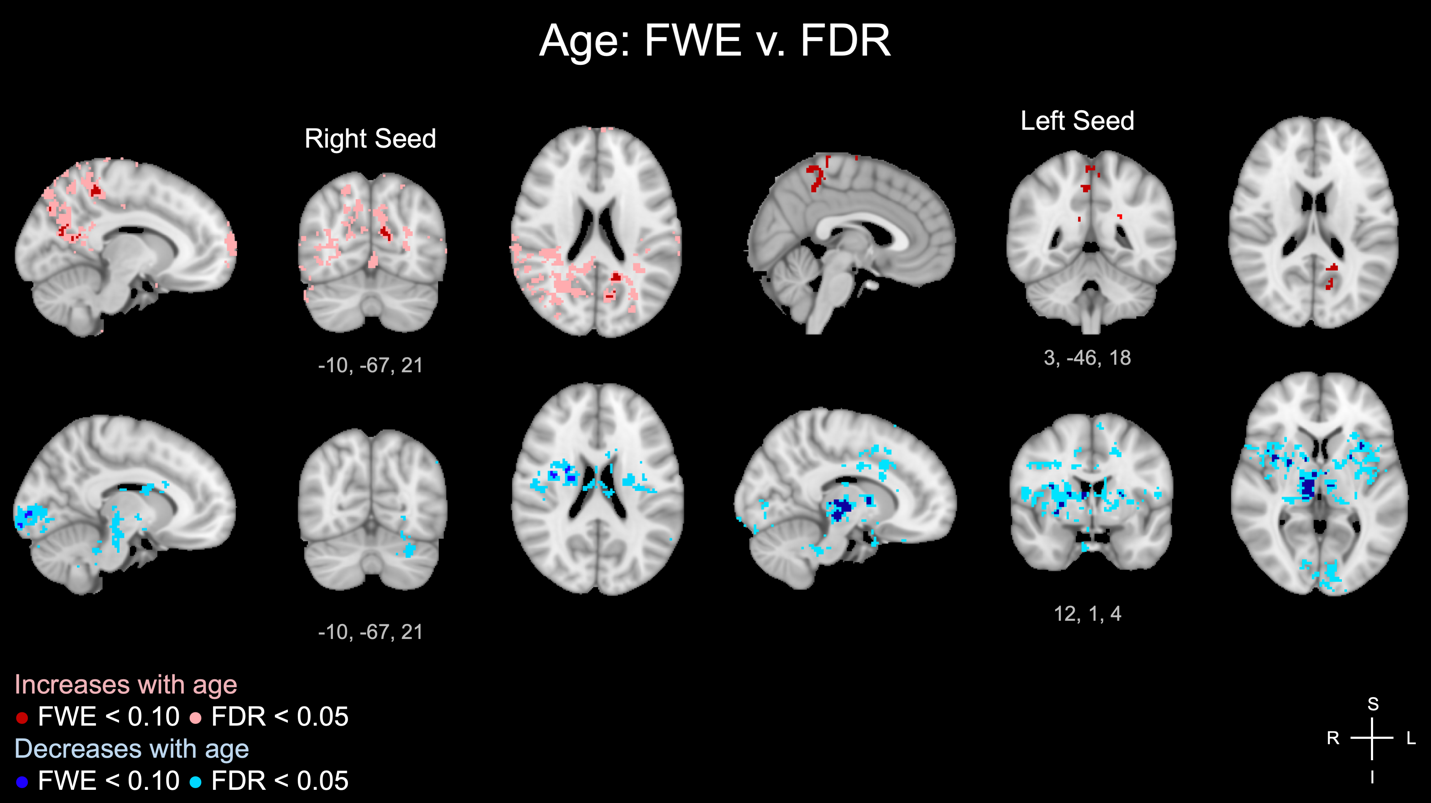


**Supplemental Figure 1.** Significant clusters in which posterior insula functional connectivity increases (red) and decreases (blue) with age are shown (using threshold-free cluster enhancement, pFWE<0.10 versus pFDR<0.05). Color intensity varies by multiple corrections approach as shown in figure legend. Clusters corresponding to the right seed are shown on the left and left seed are shown on the right. Images are shown in radiological convention.
